# Magnetoplasmonics beyond Metals: Ultrahigh Sensing Performance in Transparent Conductive Oxide Nanocrystals

**DOI:** 10.1021/acs.nanolett.2c03383

**Published:** 2022-11-08

**Authors:** Alessio Gabbani, Claudio Sangregorio, Bharat Tandon, Angshuman Nag, Massimo Gurioli, Francesco Pineider

**Affiliations:** † INSTM and Department of Chemistry and Industrial Chemistry, Università di Pisa, via G. Moruzzi 13, 56124Pisa, Italy; ‡ Department of Physics and Astronomy, Università degli Studi di Firenze, via Sansone 1, 50019Sesto Fiorentino, FI, Italy; § CNR-ICCOM, Via Madonna del Piano 10, 50019Sesto Fiorentino, FI, Italy; ∥ INSTM and Department of Chemistry “U. Schiff”, Università degli Studi di Firenze, via della Lastruccia 3, 50019Sesto Fiorentino, FI, Italy; ⊥ Department of Chemistry, Indian Institute of Science Education and Research (IISER), Pune411008, India

**Keywords:** Magnetoplasmonics, Transparent Conductive Oxides, Nanocrystals, Magneto-optics, Active Plasmonics, Sensing

## Abstract

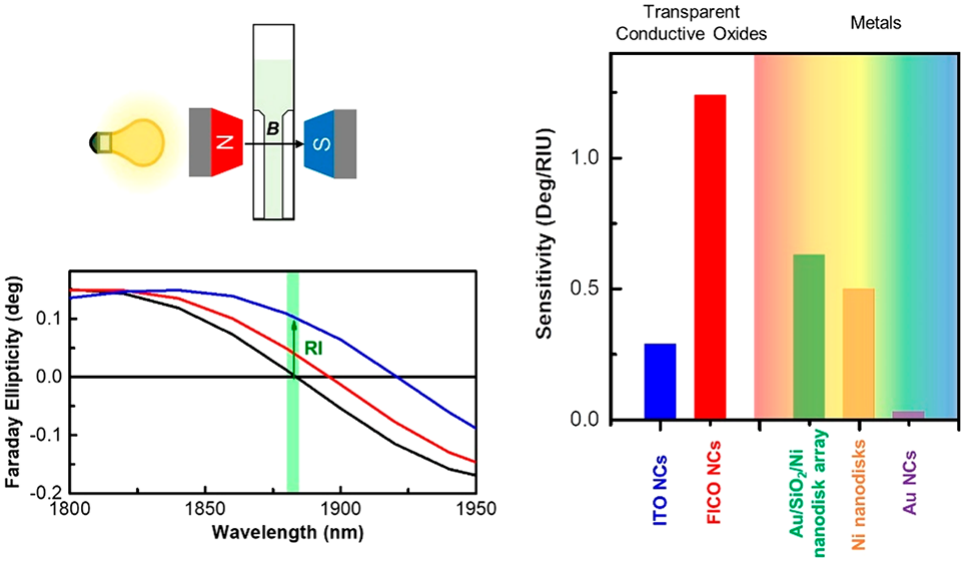

Active modulation of the plasmonic response is at the forefront of today’s research in nano-optics. For a fast and reversible modulation, external magnetic fields are among the most promising approaches. However, fundamental limitations of metals hamper the applicability of magnetoplasmonics in real-life active devices. While improved magnetic modulation is achievable using ferromagnetic or ferromagnetic-noble metal hybrid nanostructures, these suffer from severely broadened plasmonic response, ultimately decreasing their performance. Here we propose a paradigm shift in the choice of materials, demonstrating for the first time the outstanding magnetoplasmonic performance of transparent conductive oxide nanocrystals with plasmon resonance in the near-infrared. We report the highest magneto-optical response for a nonmagnetic plasmonic material employing F- and In-codoped CdO nanocrystals, due to the low carrier effective mass and the reduced plasmon line width. The performance of state-of-the-art ferromagnetic nanostructures in magnetoplasmonic refractometric sensing experiments are exceeded, challenging current best-in-class localized plasmon-based approaches.

Manipulation of light at the nanoscale is one of the great open challenges in nano-optics.^1,2^ Active plasmonics is a key player in this field, exploiting remote control of the plasmonic response to enable miniaturized, ultrafast and ultrahigh performance optical sensors based on refractometry^3−5^ or field enhancement,^6,7^ as well as tunable nanophotonic optical components.^8−11^ Several elegant approaches have been proposed to achieve active plasmonics, through controlled modification of the refractive index of the medium,^12,13^ mechanical deformation of the substrate,^14,15^ or via electric or electrochemical stimuli,^16−18^ to name a few. Unfortunately, none of these approaches satisfies the three key requirements (speed, reversibility, and ease of implementation) at the same time. To date, all-optical control of plasmon resonances have reached the highest performance in the visible and infrared range,^8,9,19−21^ even though it requires optical pumping with high power pulsed sources, which could irreversibly modify the material, making its implementation in devices challenging and expensive. On the other hand, magnetic fields are easy to generate and propagate, and their effects on charge carriers are ultrafast and fully reversible. Indeed, thanks to the advancements in magnetic storage devices, nowadays magnetic fields of the order of 1 T can be applied by the magnetic writing heads on spatial regions below 100 nm and with a switching speed of the order of the GHz.^22^ The use of magnetic fields in combination with polarized light to control plasmon resonances (magnetoplasmonics^23−25^) is thus a strong candidate for active plasmonics.

For the design of efficient active magnetoplasmonic elements, two key factors come into play: the magnitude of the magnetic modulation and the quality of the optical resonance. Using magneto-optical spectroscopic techniques, magnetically driven modulation of localized surface plasmon resonance (LSPR) has been observed on different combinations of materials: pure noble metals,^26−35^ dielectric-metallic hyperbolic nanoparticles,^36^ ferromagnetic Ni nanodisks,^37,38^ hybrid noble metal/magnetic nanostructures,^39−44^ and Au nanostructures embedded in transparent magnetic insulators.^45−47^ Nevertheless, achieving strong magnetic modulation without degrading the plasmonic properties remains challenging, and real life applications are hampered by the magnetoplasmonic trilemma: (1) a good plasmonic metal has sharp optical resonances but low magneto-optical response;^28,35^ (2) a magnetic metal has strong magnetoplasmonic response but a very broad plasmonic resonance;^38,48^ (3) mixing the two components degrades the quality of both features.^40,49^ The trilemma seems to set fundamental limitations to the development of high-performance magnetoplasmonic platforms, since an improvement in any parameter inevitably results in the deterioration of other parameters. These considerations, however, hold only for standard metals: a paradigm shift in material choice that goes beyond standard metallic plasmonic materials can indeed remove the limitations imposed by the trilemma. Indeed, novel photonic and plasmonic nanomaterials have emerged recently, such as dielectric antennas^50,51^ or heavily doped semiconductors,^52^ which have been scarcely explored for magnetoplasmonics. Here we propose transparent conductive oxides (TCOs)^53−56^ as a new class of magnetoplasmonic materials. In nonmagnetic plasmonic nanostructures, magnetic modulation can be rationalized in terms of Lorentz force acting on free charge carriers (Figure 1a). Circularly polarized light can excite circular plasmonic modes in a spherical nanoparticle, with free carriers rotating clockwise or anticlockwise depending on light helicity. The degeneracy of the two modes is removed by the application of an external magnetic field. Indeed, the latter generates a Lorentz force that has opposite sign depending on light helicity, thus causing spatial broadening or shrinking of the circular magnetoplasmonic modes. The magnetic field thus acts as a perturbation on the free carrier motion, resulting in a helicity-dependent red or blue shift of the plasmonic resonance condition, modifying the imaginary and real part of the polarizability of the plasmonic sphere as depicted in Figure 1b,c, leading to the emergence of Faraday ellipticity and rotation, respectively. The magnitude of the magneto-optical signal (ellipticity and rotation) is proportional to the energy shift between the circular magnetoplasmonic modes, which in first approximation is proportional to the cyclotron frequency (*ω*_c_ = *eB*/*m*),^28,34^ where *e* and *m* are the charge and effective mass of electrons, respectively, and *B* is the applied magnetic field (assuming zero magnetization of the material). A crucial parameter to improve the magneto-optical response is thus represented by *m* (Figure 1d).

**Figure 1:**
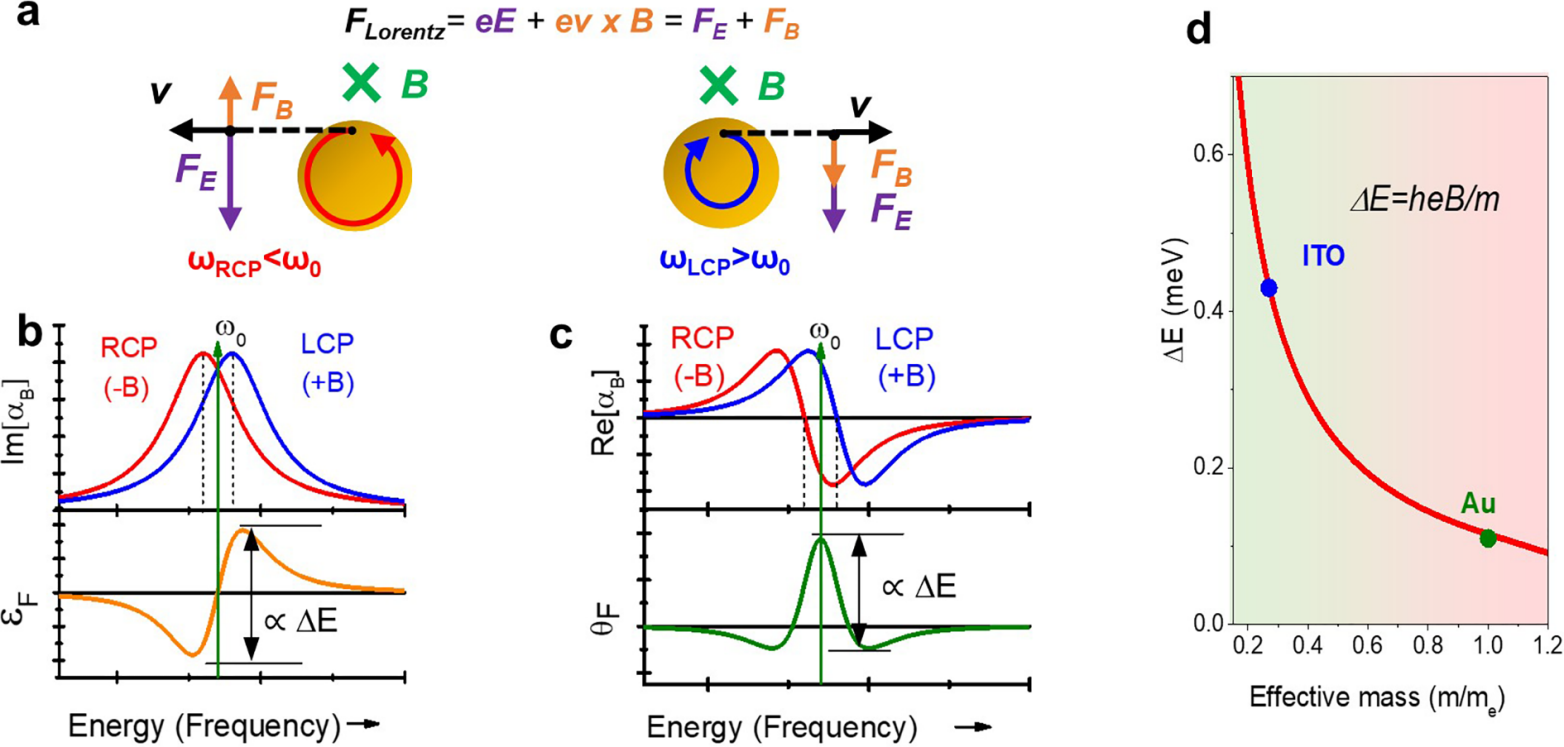
(a) Magnetic field induced modification of the Lorentz force acting on free electrons in a plasmonic nanoparticle. In the presence of an external magnetic field, a change in the helicity of incoming light reverses the direction of the magnetic component of the Lorentz force, thus inducing a change in the frequency of the free electron oscillation. Two circular magnetoplasmonic modes can thus be excited by RCP or LCP light in the presence of an external magnetic field (B). (b,c) Due to modification of the Lorentz force, the imaginary part (b) and the real part (c) of the field-dependent NP polarizability (α_B_) are modified, thus leading to the emergence of Faraday ellipticity ε_F_ (b) and Faraday rotation θ_F_ (c), respectively. The magnitude of ε_F_ and θ_F_ is proportional to the magnetic field induced energy shift (Δ*E*). (d) Relation between Δ*E* and free electron effective mass, highlighting the importance of using materials with low effective mass to improve the magnetoplasmonic effect.

For most metals *m* has a fixed value, close to the free electron mass *m*_e_; conversely, in heavily doped semiconductors, carrier parameters (charge, density and mass) can be modulated by doping.^57−62^ TCOs display reduced carrier effective mass (*m*) with respect to noble metals (down to 0.4–0.2*m*_e_),^57,58^ which makes them very attractive for magnetoplasmonics. Nevertheless, the application of TCO nanostructures in this field has not been explored to date.

In this work, employing colloidal dispersions of TCO nanocrystals (NCs) we achieved a magnetoplasmonic modulation which is unprecedented in nonmagnetic plasmonic materials and is strongly competitive with state-of-the-art magnetic-plasmonic architectures, while retaining superior resonance sharpness. These features prompt improved refractometric sensing platforms with performance indexes challenging both optical and magneto-optical approaches. Monitoring the Faraday ellipticity of F- and In-doped CdO (FICO) NCs at fixed wavelength affords a sensitivity of 1.24 deg per refractive index unit, potentially detecting refractive index changes down to 3 × 10^–6^, without requiring a fitting approach. We believe that these results open up a new route toward high performance magnetoplasmonic technologies.

Among TCOs, Sn-doped In_2_O_3_ (ITO) and FICO NCs have been chosen: the first one is the most established TCO and has a superior magneto-optical response compared to noble metal NCs;^52^ the second one has a significantly reduced LSPR line width, thanks to cooperative anion–cation codoping,^63,64^ which is expected to further improve the magneto-optical response. Carriers in both semiconductors have a relatively small electron effective mass (≈0.3–0.4*m*_e_).^52,65^

Quasi-spherical ITO and FICO NCs with plasmonic resonance in the same near-infrared wavelength range were synthesized by colloidal chemistry approaches^63,66^ (for more details see section 1 of the Supporting Information file). Transmission electron microscopy (TEM) morphological and structural analysis revealed a mean size of 9.0 ± 1.7 nm and 15.0 ± 2.3 nm for ITO and FICO NCs, respectively (Figure 2a,b and Figure S1). The X-ray diffraction peaks (Figure 2c) show negligible shifts with respect to those of undoped In_2_O_3_ and CdO, respectively, without secondary crystalline phases (Cu Kα radiation was used as the X-ray source). The aliovalent dopant incorporation was confirmed by Inductively Coupled Plasma Atomic Emission Spectroscopy (ICP-AES), revealing a content of 10% of Sn and 26% of In for ITO and FICO NCs, respectively. The optical extinction spectra collected in a CCl_4_ dispersion (Figure 3a,b) revealed the presence of an electric dipole plasmonic resonance (with negligible scattering contribution due to the reduced NCs size compared to the wavelength of the impinging light) at near-infrared wavelengths with peaks at 1825 nm (0.679 eV) and 1883 nm (0.659 eV) for ITO and FICO NCs, respectively. The LSPR peaks of the two NCs display significantly different full-widths at half-maximum (fwhm), which are 0.19 and 0.10 eV, respectively, for ITO and FICO NCs. This difference is justified by the different electron scattering with dopant impurities in the two semiconductors.^63−65,67^ No effect of the NCs size and polydispersity on the LSPR line width is predicted by Mie theory given the small diameter of the NCs compared to incident wavelength.^68,69^ However, ensemble heterogeneities may increase the plasmonic line width in ensemble spectra generating a distribution of plasma frequencies and causing small shifts of the resonance condition. This was found by Johns et al. experimentally using single particle FTIR spectroscopy on ITO and AZO NCs with LSPR in the infrared,^70^ revealing that heterogeneous dopant incorporation is the dominant contribution, with also a minor effect of shape inhomogeneities. Such shape heterogeneity is slightly present in our ITO NCs, as elongation of some NCs can be seen also in Figure 2a, resulting in the presence of multiple plasmonic modes close in energy, while it seems less important in FICO NCs (Figure 2b). FICO NCs display reduced LSPR damping, providing sharper LSPR peaks than ITO and a higher quality factor of the resonance (6.6 vs 3.6).

**Figure 2:**
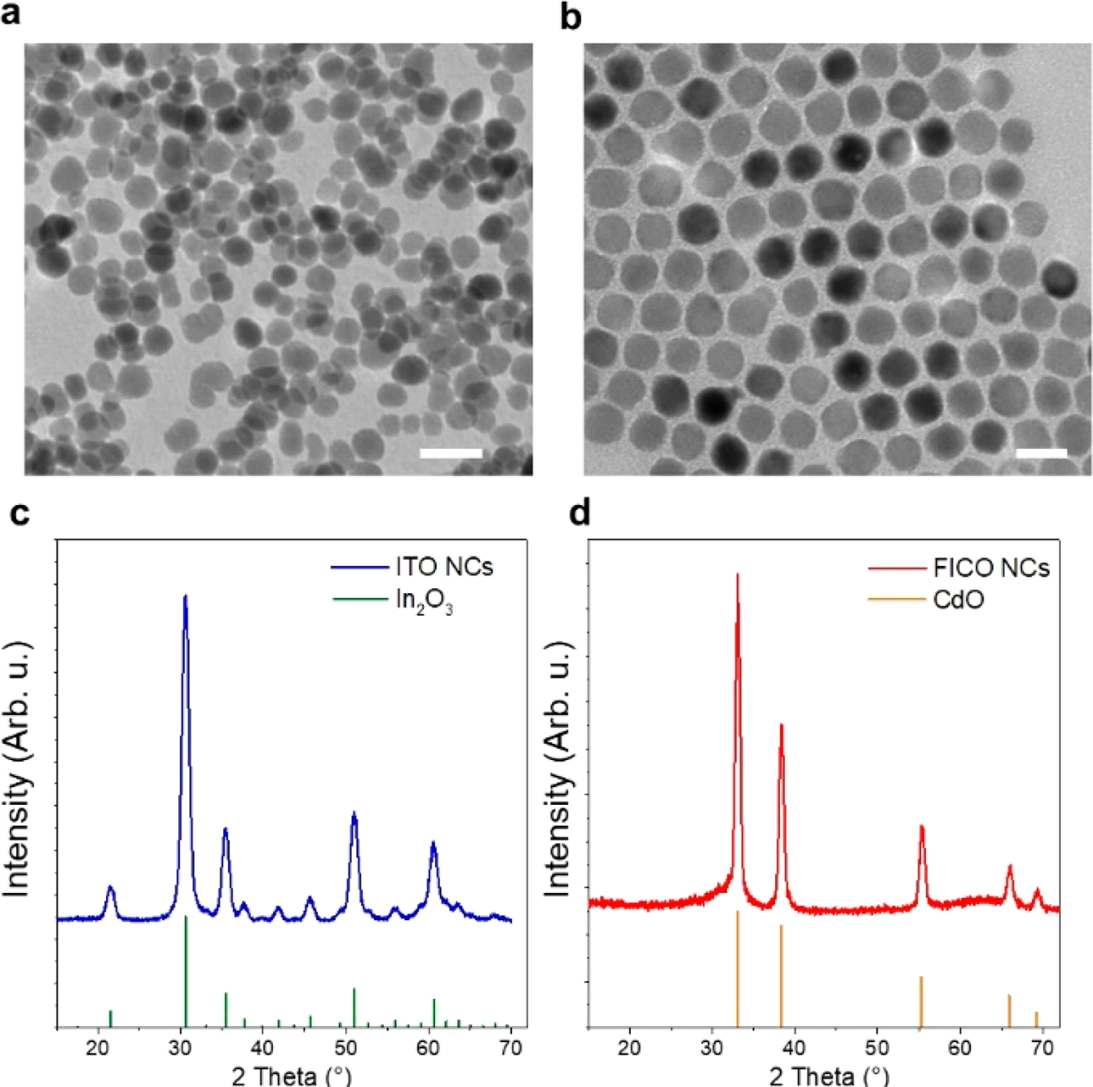
Morphology and crystal structure of ITO and FICO colloidal nanocrystals. Representative TEM images of ITO (a) and FICO NCs (b). Scale bar is 20 nm. Powder X-ray diffraction pattern collected for ITO (c) and FICO NCs (d), using the Cu Kα radiation (1.54 Å) as the X-ray source. The reference patterns of In_2_O_3_ (PDF 06-0416) and CdO (PDF 05-0640) are shown for comparison.

**Figure 3:**
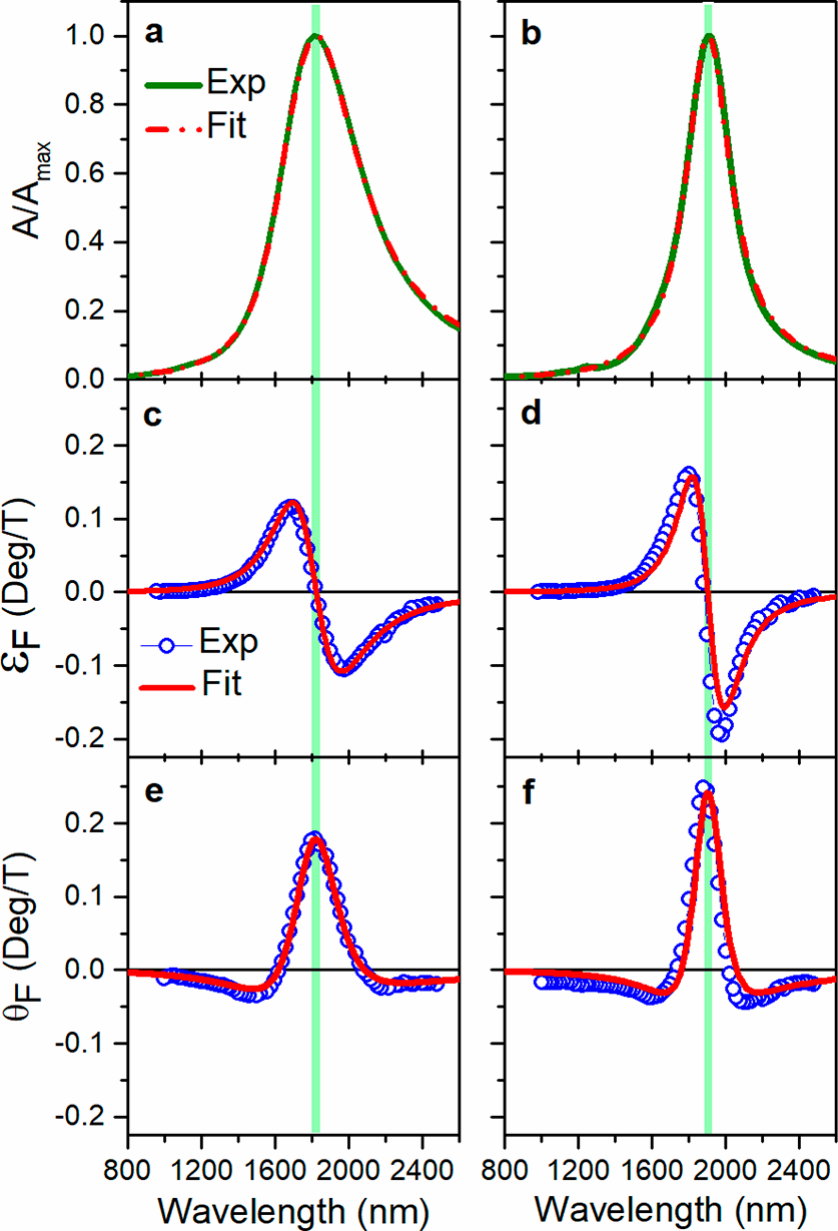
Optical and magneto-optical properties. Normalized experimental extinction spectra (*A*/*A*_max_) of ITO (a) and FICO (b) NCs dispersed in CCl_4_ are reported in green, together with the fitting curve (red line); (c,d) corresponding experimental Faraday ellipticity (*ε*_F_, blue dots) and fitting curve (red line); (e,f) experimental Faraday rotation (*θ*_F_, blue dots) of the same samples, and corresponding fitting curve (red line). The magneto-optical response is normalized to the extinction maximum for the sake of comparison and reported in deg for unit (Tesla) of applied field. The measurements were carried out at room temperature. The vertical pale green line indicates the LSPR wavelength.

The full magneto-optical response was investigated at room temperature by measuring the Faraday rotation (*θ*_F_) and ellipticity (*ε*_F_) spectra of the NCs dispersed in CCl_4_ (Figure 3c,d). For a better comparison between the two samples, the MO signal is normalized for the optical density of the dispersion and for the applied magnetic field. *ε*_F_ features a dispersive shape, crossing the zero at the LSPR energy (*E*_0_). This is consistent with a helicity-dependent opposite shift (to higher/lower energy for LCP/RCP excitation respectively) of the resonance condition with respect to *E*_0_, which is driven by the applied magnetic field. On the other hand, *θ*_F_ displays a positive maximum at the LSPR energy, with two weaker negative peaks, one at higher energy and the other at lower energy with respect to *E*_0_. Both magneto-optical signals show a linear magnetic field dependence, as expected for nonmagnetic plasmonic nanostructures.^28,34,35^

The optical response of plasmonic nanospheres can be calculated from the quasi-static polarizability of a sphere (α).^68,69^ The effect of magnetic field on LSPR can be rationalized in terms of field-driven modification of the NC polarizability (eq S4 and ref (71)), resulting in the splitting of the otherwise degenerate circular plasmonic modes (Figure 1a), as already reported for noble metal nanostructures.^28,31,71^ The magnetic field splitting of the circular magnetoplasmonic modes modifies the real and imaginary parts of the polarizability, thus leading to Faraday rotation and ellipticity respectively (Figure 1b,c). An analytical expression for the helicity-dependent differential polarizability is thus obtained (under an applied magnetic field) (*Δα*_B_(ω) = α_LCP_(ω) – α_RCP_(ω)). From this expression, *θ*_F_ and *ε*_F_ can be calculated through eqs 1 and 2 (see section 3 of the Supporting Information for more details).
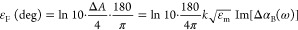
1
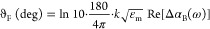
2where *k* is the wavevector of light, ε_m_ is the solvent permittivity, and (ln 10·180/4π) is the conversion factor of the signal from MCD (Δ*A* units) into ellipticity angle (degrees).^72^

The dielectric functions of ITO and FICO can be expressed in terms of the carrier parameters *N*, *m*, and γ, according to the Drude model (see section S2 of the Supporting Information for details)^67,69^ and inserted into the quasi-static field-dependent polarizability (eq S4) to obtain the extinction cross section of the NCs. Then, using the analytical equations, we extract the fundamental parameters of the charge carriers involved in LSPR, i.e., carrier density *N*, mass *m*, and damping parameter γ, through the simultaneous fitting of the normalized ellipticity, rotation, and extinction spectra (more details are provided in section S2 of the Supporting Information). The determination of the Drude parameters is critical, as they are the main factors affecting the optical and magneto-optical response. The ratio *N*/*m* controls the LSPR position, whereas *m* is inversely proportional to the magnetic modulation of LSPR. The obtained Drude parameters are reported in Table S1. Remarkably, Faraday ellipticity and rotation are in excellent agreement with the analytical model employed (Figure 3c–f). Carrier densities of 7.00 × 10^20^ and 8.96 × 10^20^ cm^–3^ were obtained for ITO and FICO NCs, respectively, roughly 2 orders of magnitude lower than Au. The comparison with Au NCs (Table S1) also shows that the reduced values of effective mass (0.26*m*_e_ and 0.30*m*_e_ for ITO and FICO NCs, respectively) are the main cause for the significantly boosted magneto-optical signal (up to 40-fold) in transparent conductive oxide NCs.

An important role is also played by the broadening of the LSPR peak (in first approximation related to γ): this is clearly shown by the stronger magneto-optical signal of FICO with respect to ITO NCs. In fact, the two materials display comparable effective mass but different LSPR line width. These findings highlight the two fundamental requirements to boost magnetoplasmonic modulation in nonmagnetic plasmonic NCs: high cyclotron frequency (achievable in materials with low electron effective mass) and reduced LSPR line width. A magnetoplasmonic quality factor can thus be defined as the ratio between cyclotron frequency and LSPR line width (*ω*_c_/γ), reported in Table S1 for the TCO NCs of this study compared to Au colloidal NCs.

To our knowledge, this is the highest magneto-optical response for a nonmagnetic plasmonic nanostructure, reaching values close to some ferromagnetic or hybrid noble metal–ferromagnetic nanostructures at room temperature and relatively low magnetic fields (1 T). Indeed, signals of 0.15–0.28 deg were obtained for FICO NCs, which are close to those reported for Ni nanodisks (∼0.5 deg at 0.4 T)^37^ and larger than those of Au/Co/Au sandwich nanodisks structures (0.1–0.2 deg at 1 T).^40^ The sharp features in the magneto-optical spectrum of FICO NCs are interesting for sensing, because a steeply sloping signal at the resonance condition will boost the sensitivity of refractometric sensors. Inspired by the above considerations, we performed a proof of concept refractometric sensing experiment by dispersing the NCs in three solvents that are transparent and have different refractive indexes (RI) in the spectral range of interest (Figure S4): CCl_4_ (RI = 1.4477), C_2_Cl_4_ (RI = 1.4895), and CS_2_ (RI = 1.5866). Extinction, Faraday rotation, and ellipticity spectra of the NCs dispersions were measured for both FICO and ITO NCs.

The RI sensitivity obtained (Figure S5) by tracking the shift of the LSPR wavelength is larger than Au nanospheres and comparable to ferromagnetic magnetoplasmonic systems (Table S3). The positive peak of the Faraday rotation spectrum (Figure 4a,b) and the zero crossing of the ellipticity (Figure 4c,d) also red shift with the increasing refractive index of the medium. Moreover, monitoring the change in intensity of the magneto-optical signal at a fixed wavelength, a drastic variation with RI can be observed, as the resonance is shifted and the slope of the signal (*δε*_F_/*δλ* and *δθ*_F_/*δλ*) is very high due to the large cyclotron frequency and the reduced LSPR line width. In Figure 4e,f, the variation of ellipticity and rotation as a function of RI is reported at a fixed wavelength (indicated by the vertical green line in Figure 4a–d) for an applied field of 1.4 T. The linear fit (dashed line in Figure 4e,f) of the MO signals collected at fixed wavelength and in the three different solvents allows calculating the RI sensitivity as the slope of the fit: *δε*_F_/δRI and *δθ*_F_/δRI. The sensitivity is compared in Figure 4e,f with the values achieved in the literature for the state-of-the-art magnetoplasmonic nanostructures (summarized in Table S3). ITO and FICO NCs showed a sensitivity of 0.29 and 1.24 deg/RIU, respectively, using the ellipticity signal, whereas values of 0.22 and 1.12 deg/RIU are obtained using the rotation signal. The best performance is displayed by the ellipticity signal of FICO NCs, reaching a RI sensitivity which is 40-fold higher with respect to our previous work on Au NPs,^28^ 2.5-fold higher with respect to Ni nanodisks,^37^ and superior to Au/SiO_2_/Ni multilayered nanodisks arranged in a periodic array exploiting surface lattice resonance to boost the sensitivity (Figure 4e,f and Table S3).^42^

**Figure 4:**
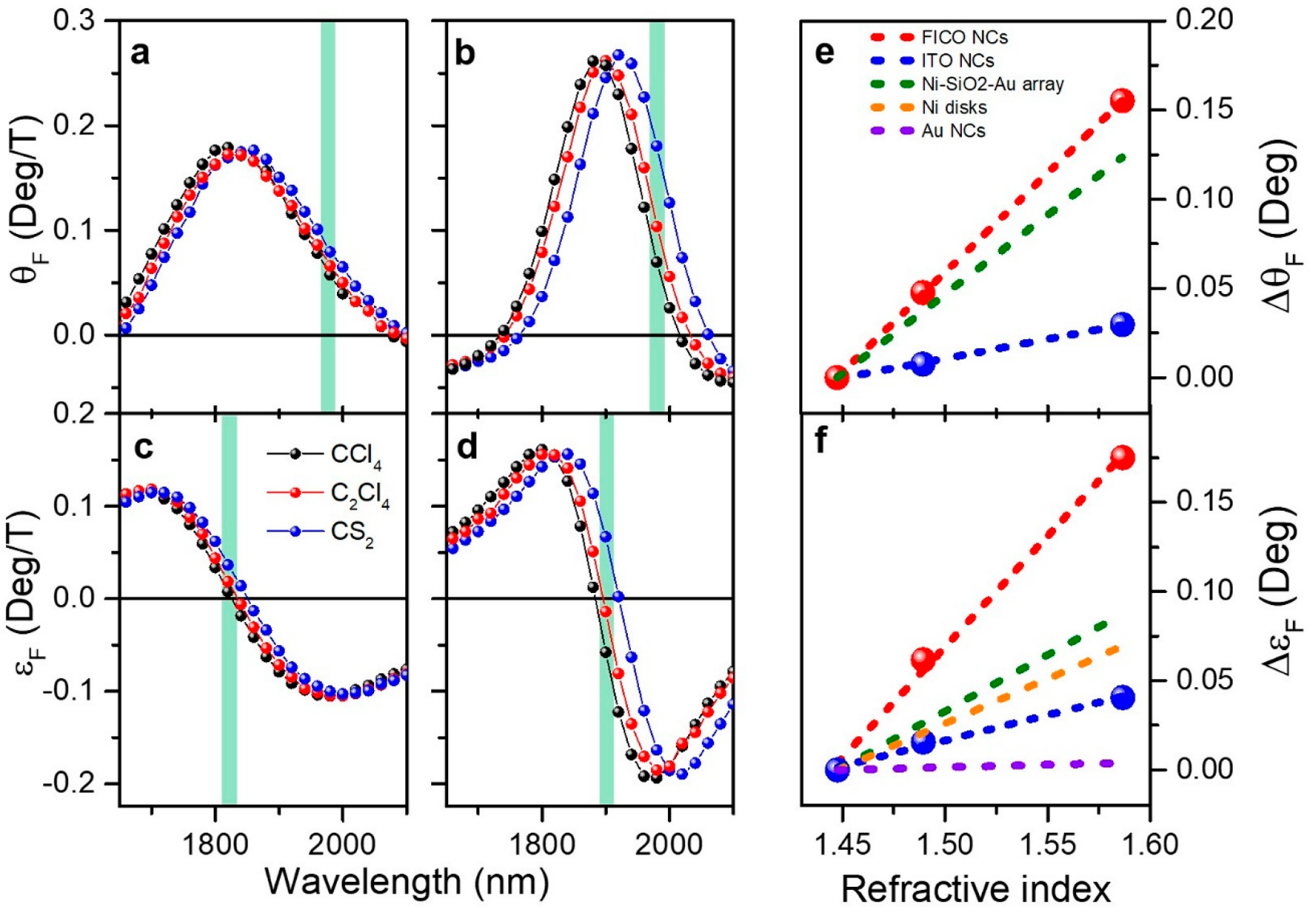
Mangetoplasmonic refractometric sensing experiment using ITO and FICO NCs. Normalized Faraday rotation *θ*_F_ (a,b) and ellipticity *ε*_F_ (c,d) spectra of ITO and FICO NCs dispersed in CCl_4_ (black line), C_2_Cl_4_ (red line), and CS_2_ (blue line). The variation of *θ*_F_ and *ε*_F_ with the refractive index at a fixed wavelength, highlighted by the vertical green line in (a–d), for 1.4 T of applied field is plotted in (e) and (f) respectively, where the dots represent the experimental points, while the dashed line is the linear fit. The errors on the ellipticity and rotation signals are smaller than the data points. The values reported in the literature for Au spherical colloidal NCs,^28^ Ni nanodisks,^37^ and Au/SiO_2_/Ni nanodisks array^42^ are reported for comparison as dashed purple, orange, and green lines, respectively (details on the experimental conditions, i.e., applied field and wavelength range, are reported in Table S3).

The enhanced RI sensitivity achieved with FICO NCs is even more remarkable if we consider that it has been obtained with simple colloidal NCs, without requiring a complex multicomponent architecture. On the other hand, ITO NCs achieved lower sensitivity, due to the reduced slope of the magneto-optical signal at the resonance condition. This can be ascribed to the decreased LSPR quality factor of ITO due to the higher electron scattering by ionic impurities, pointing out the importance of sharp resonances for this sensing approach.

In view of device applicability, a remark should be made on the magnetic field dependence of magnetoplasmonic effects. In ferromagnetic materials,^37,40^ the magnitude of the MO signal is proportional to their magnetization. In nickel nanodisks, for instance, the magneto-optical signal saturates at ≈0.4 T; a higher field value will not yield any signal increase. In nonmagnetic TCOs, on the other hand, MO effects scale linearly with the applied field. This can be an advantage or a disadvantage (Ni can be saturated with a cheap NdFeB permanent magnet). However, with proper miniaturization, high magnetic field values can be achieved in portable devices (hard drive write heads apply fields up to 2.4 T^73^), giving a net advantage to nonmagnetic TCOs.

Considering that our setup is able to measure signals down to 0.33 mdeg (1 × 10^–5^ Δ*A* units, i.e., three times the standard deviation of the measurement), ΔRI down to 2.6 × 10^–4^ can be easily detected. A magneto-optical setup specifically optimized for high sensitivity can detect signals down to 3 × 10^–6^ mdeg (1 × 10^–7^ Δ*A* units),^74^ affording RI sensitivities of 3 × 10^–6^, close to the current technologies that employ the tracking of LSPR maximum measured in extinction spectroscopy.^75,76^ A limitation of the latter approach lies in the fact that curve fitting procedures are generally required to detect very small shifts of the wide LSPR resonance and the method can become unstable in a real analytical matrix. Our detection strategy, on the other hand, does not require a fitting procedure, but simply consists of measuring changes in the intensity of the Faraday ellipticity at a fixed wavelength. Even if single wavelength measurements are potentially achievable also in extinction spectroscopy mode, we believe that the strongly sloping signal of MO ellipticity provides an advantage for single wavelength detection with respect to tracking intensity variations at fixed wavelength close to the LSPR maximum in extinction spectroscopy. In addition to the high sensitivity afforded by the sloping magneto-optical signal, our approach has the advantage of using an observable that is modulated both in polarization and in magnetic field, thus making it very stable to matrix-related interference: virtually any interference (even chiral molecules, but with the exception of magneto-optically active species) will be filtered out by this dual modulation.

In conclusion, nonmagnetic ITO and FICO NCs display exceptional magneto-optical response, challenging state-of-the-art ferromagnetic magnetoplasmonic nanomaterials.^37,40,42^ This is afforded by the simultaneous presence of sharp plasmon resonances and lower electron effective mass with respect to metals. The experimental spectra are in qualitative and quantitative agreement with our analytical model based on circular magnetoplasmonic modes. Proof of concept experiments demonstrate the applicability of the investigated NCs for magnetoplasmonic refractometric sensing, with dramatically improved performance compared to Au NCs and even superior with respect to the most promising magnetoplasmonic systems reported in the literature based on magnetic metals. Moreover, the sensitivity of our proposed approach is competitive with the current state-of-the-art of refractometric sensing based on LSPR measured in extinction spectroscopy, with the advantage of not relying on a fitting procedure.

Considering the current growing interest in semiconductor NCs, in the near future the advancement in their synthesis and in the understanding of the correlation between structural parameters and the optical response could potentially lead to even sharper plasmonic resonances which could further increase the sensitivity of our proposed approach. We believe that our understanding of the magneto-optical response in heavily doped semiconductor NCs can trigger a new interest in these materials for magnetoplasmonics and in closely related topics such as light-induced magnetism in plasmonic nanoparticles^77^ and plasmon-induced carrier polarization.^78^ To this aim, we can envisage heavily doped semiconductor NCs as building blocks for hybrid nanostructures, by combining them with a magnetic unit^79^ or codoping them with magnetic ions.^53,80−82^

## Supporting Information Available

Detailed experimental methods section; size distribution of the nanocrystals obtained from the analysis of TEM images; further details on the analytical model and fitting procedures employed; refractive index of the solvents employed in the experiments as a function of the wavelength; extinction spectra of ITO and FICO NCs dispersed in CCl_4_, C_2_Cl_4_, and CS_2_; table summarizing the magnetoplasmonic performances of FICO and ITO NCs compared with the most promising magnetoplasmonic systems reported in the literature (PDF)
