# Supplementary material for: Magnetoplasmonics beyond Metals: Ultrahigh Sensing Performance in Transparent Conductive Oxide Nanocrystals
Source: Nano Lett. 2022 Nov 8;22(22):9036–44. doi: 10.1021/acs.nanolett.2c03383 (PMC9706655; doi:10.1021/acs.nanolett.2c03383)
Supplement: Supplementary file 1 — nl2c03383_si_001.pdf [file nl2c03383_si_001.pdf]

Supporting Information

Magnetoplasmonics Beyond Metals: Ultrahigh Sensing Performance in Transparent Conductive Oxide Nanocrystals

Alessio Gabbani,[1,2,3]* Claudio Sangregorio,[3,4] Bharat Tandon,[5] Angshuman Nag,[5] Massimo Gurioli,[2] Francesco Pineider[1,2]*

1 INSTM and Department of Chemistry and Industrial Chemistry, Università di Pisa, via G. Moruzzi 13, 56124, Pisa Italy

2 Department of Physics and Astronomy, Università degli Studi di Firenze, via Sansone 1, 50019 Sesto Fiorentino, FI, Italy

3 CNR-ICCOM, Via Madonna del Piano 10, 50019 Sesto Fiorentino (FI), Italy

4 INSTM and Department of Chemistry "U. Schiff", Università degli Studi di Firenze, via della Lastruccia 3, 50019 Sesto Fiorentino (FI), Italy

5 Department of Chemistry, Indian Institute of Science Education and Research (IISER), Pune 411008, India

***Corresponding Authors**

alessio.gabbani@dcci.unipi.it

francesco.pineider@unipi.it

S1. Methods

Synthesis of ITO NCs. 1.8 mmol of $In(acac)_3$ and 0.2 mmol of $Sn(acac)_2Cl_2$ were dissolved in 20 mL of 1-octadecene with 6 mmol of oleylamine and 6 mmol of oleic acid. Under constant magnetic stirring and under vacuum the reaction mixture was heated to 80°C over 10 min and maintained for 30 min. Then, under a nitrogen atmosphere, the temperature was increased to 160°C over 12 min and maintained for 1h, after which it was increased to 310°C over 26 min and maintained for additional 2h. The mixture was cooled down to room temperature by removing the heat source. The NCs were washed twice by centrifugation in ethanol at 4400 g for 5 minutes, after which they were dispersed in hexane.

Synthesis of FICO NCs. The synthetic procedure was adapted from Ye *et al.*[57] 1.2 mmol of $Cd(acac)_2$ and 0.3 mmol of InF_3 were added to 5.25 mmol of oleic acid and 50 mL of 1-octadecene. The mixture was heated to 120 °C for 1h under vacuum, after which the temperature was raised to reflux (320°C) during 18 min under Ar atmosphere. After 15 min at reflux the mixture changed color to dark green, and after additional 10 min the reaction was stopped by removing the heating source and the mixture was cooled down to room temperature. The NCs were washed twice by centrifugation in isopropanol at 4400 g and dispersion in hexane. Bulky secondary products were removed by centrifugation in hexane at 1000 g and discarding the bulky precipitate.

XRD. Powder X-ray Diffraction (XRD) measurements were carried out using a Bruker D8 Advance diffractometer equipped with a Cu K_α radiation and operating in Theta-Theta Bragg Brentano geometry at 40 kV and 40 mA.

TEM. Transmission Electron Microscopy (TEM) analysis was performed using a JEOL 100 SX, operating at 100 kV. Samples were prepared by drop drying a dilute suspension of NCs in hexane onto 200 mesh carbon-coated copper grids. The mean size and size distribution were obtained from statistical analysis over at least 300 NCs.

ICP-AES. ICP-AES measurements were performed in triplicate by using a Varian 720-ES Inductively Coupled Plasma Atomic Emission Spectrometer, on samples (≈1 mg) digested in concentrated aqua regia (HCl suprapure and HNO3 sub-boiled in 3:1 ratio) and in the presence of H_2O_2, diluted with ultrapure water (≥18 MΩ) and analyzed using Ge as internal standard. Calibration standards were prepared by gravimetric serial dilution from monostandard at 1000 mg/L. The wavelengths used for In, Sn and Cd were 325.6, 189.9 nm and 214.4 nm respectively. The dopant content is defined as $n_D/(n_D+n_C)\cdot 100$, where n_D and n_C are the moles of substitutional dopant and lattice cation respectively.

UV-vis-NIR Extinction measurements. Extinction spectra were collected using a commercial Cary5000 UV-vis-NIR spectrophotometer (Agilent), using a 1 mm quartz QX cuvettes. Solvents transparent in the NIR (CCl_4, C_2Cl_4 and CS_2) were used to disperse the NCs for optical and magneto-optical characterization.

Magneto-optical characterization. Magneto-optical characterization was performed in the Faraday configuration using in house built MO set up in the spectral range 800-2500 nm, and exploiting the polarization modulation technique using a photo-elastic modulator (PEM) which modulates the light polarization alternatively between RCP and LCP at a frequency of 47 kHz. Light provided by the Xe lamp (power 300 W) passes through a monochromator (Oriel Cornerstone 260) and then is linearly polarized at 90° using a Rochon polarizer. Linearly polarized light is then focused on the sample which is placed in an electromagnet able to apply a magnetic field of 1.4 Tesla. The polarization of light transmitted by the sample is then modulated by the PEM, whose retardation is set to 0.383λ, after which an analyser (Glan-Thompson prism) oriented at 45° is placed before an InGaAs detector. The output from the detector is analysed by two lock-in amplifiers, locked respectively to the first and the second harmonic of the PEM, collecting the signal related to Magnetic Circular Dichroism or Faraday ellipticity (ε_F) and Faraday rotation (θ_F) respectively. To filter out the residual environmental light, the signal is further modulated at 440 Hz by a mechanical

chopper. A third lock-in amplifier locked to the chopper is used to retrieve the total light collected by the detector. The MO signal is calibrated using a $K_3Fe(CN)_6$ solution as a standard reference sample,[72] in order to calibrate the ratio between the AC and the DC signal that reaches the detector. A Hall probe is employed to measure the effective magnetic field in the sample position. The spurious dichroic signal of the set up was subtracted by measuring the MO signals at +1.4 Tesla and -1.4 Tesla: the semi-difference between the two gives the MO signal, assuming that the natural dichroism and optical rotation are invariant with the magnetic field. The measurements were performed in solution, using 1 mm quartz QX cuvettes. The Faraday rotation and ellipticity of the solvent were collected in the same experimental conditions and subtracted from the signal of the sample, in order to isolate the contribution of the NCs. The MO signal was then normalized for the optical density and for the applied magnetic field, and converted into ellipticity and rotation angles (in degrees) according to previous work.[64,66] The optical density of the NC dispersions analysed was in the range 0.7-1.4, which is in the optimal range that maximizes the signal-to-noise ratio.[64] The MO spectra reported in the main text are collected at 1.4 Tesla, and normalized for the optical density and the applied magnetic field.

Analytical Calculation of the optical and magneto-optical response. The calculation of the optical and magneto-optical response was performed with an analytical model previously developed.[27,63] The Drude dielectric function of the semiconductors was inserted in the quasi-static field- and helicity-dependent polarizability (equation S 4). The fitting of extinction and ellipticity spectra was performed using the same analytical model, extracting carrier parameters N, m and γ. More details are provided in section S2.

S2. Structural characterization

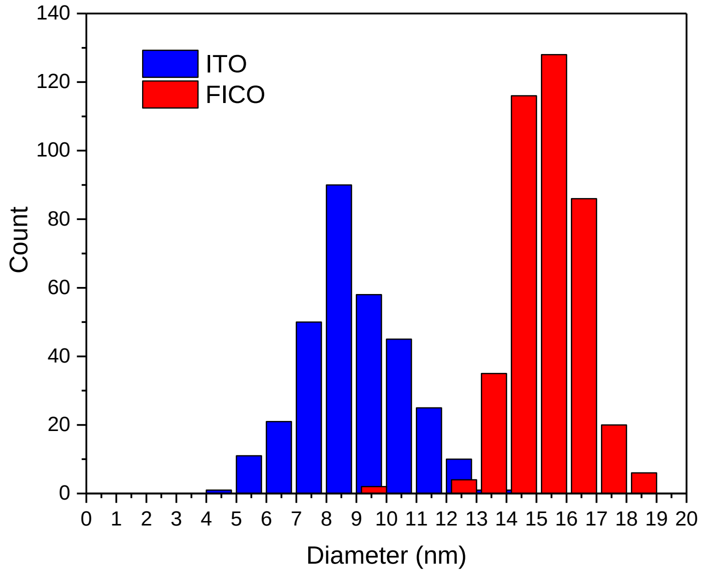

Figure S 1: Size distribution of ITO and FICO NCs, determined from TEM statistics.

S3. Analitical model and fitting of extinction and magneto-optical spectra

To fit the normalized extinction, Faraday ellipticity and rotation spectra, a matlab routine was developed, according to an analytical model introduced in previous work.[1,2]

In the dipolar quasi-static approximation, the polarizability of a spherical NC of size D and dielectric function $\varepsilon(\omega)$ in a dielectric medium with dielectric constant ε_m, can be expressed through Equation S 1, and can be related to the extinction cross section (Equation S 2):[3]

Equation S 1 $\alpha(\omega) = -\frac{\pi D^3}{2}\frac{(\varepsilon(\omega)-\varepsilon_m)}{(\varepsilon(\omega)+2\varepsilon_m)}$

Equation S 2 $\sigma(\omega) = k\sqrt{\varepsilon_m} Im[\alpha(\omega)]$

MO effects in purely plasmonic NCs are rationalized in terms of the Lorentz force acting on free electrons:[2,4,5]

Equation S 3 $m\frac{dv}{dt} + \gamma m v = -eE - ev \times B$

where e and m are the charge and effective mass of the electron, v its velocity, γ is the damping parameter and B is the external magnetic induction. Commonly, the effect of the magnetic field on the dielectric function of a metal is treated adding an off-diagonal term in the dielectric tensor or in the polarizability tensor, which takes into account for the magnetic-field driven modification of the dielectric function.[4,6] Alternatively, the problem can be also solved by using a diagonal form of the polarizability, as reported by Gu and Kornev,[1] who, exploiting the fact that the term $ev \times B$ is smaller with respect to the other term, solved Equation S 3 perturbatively. Following the formulation by Gu and Kornev, the field- and helicity-dependent polarizability can be written in a diagonal form according to Equation S 4:[1,2]

Equation S 4 $\alpha_B(\omega) = -\frac{\pi D^3}{2}\frac{(\varepsilon(\omega)-\varepsilon_m)+B(f(\omega)-f_m)}{(\varepsilon(\omega)+2\varepsilon_m)+B(f(\omega)-f_m)}$

where the second term at the numerator and denominator describes the effect of the magnetic field on the polarizability, in which $f(\omega) = f_1 + if_2$ and f_m are the coupling functions describing the interaction with the magnetic field for the NC and the medium. At zero applied magnetic field, Equation S 4 is simplified to the well-known quasi-static polarizability of a sphere (Equation S 1).

Considering the symmetry of the problem, a change in helicity is equivalent to an inversion of the direction of the external magnetic field B. It follows that the differential polarizability ($\Delta\alpha_B(\omega) = \alpha_{LCP}(\omega) - \alpha_{RCP}(\omega)$) can be obtained from the difference between the polarizability calculated with positive and negative applied field. Using the obtained $\Delta\alpha_B$, the helicity-dependent normalized differential cross section can be readily obtained through Equation S 5.

Equation S 5 $\Delta\sigma_{norm}(\omega) = \frac{\Delta\sigma(\omega)}{\sigma_{max}} = \frac{k\sqrt{\varepsilon_m}Im[\Delta\alpha_B(\omega)]}{\sigma_{max}}$

The $\Delta\sigma$ can be converted from differential absorption units into ellipticity angle (ε_F) using Equation S 7. The following equations were thus employed to fit the normalized extinction and Faraday ellipticity spectra:

Equation S 6 $\sigma_{norm}(\omega) = \frac{\sigma(\omega)}{\sigma_{max}} = \frac{k\sqrt{\varepsilon_m}Im[\alpha(\omega)]}{\sigma_{max}}$

Equation S 7 $\varepsilon_F\,(deg) = ln10 \cdot \frac{\Delta\sigma_{norm}(\omega)}{4} \cdot \frac{180}{\pi}$

Similarly, an equation for the fitting of the Faraday rotation spectra can be derived:

Equation S 8 $\vartheta_F\,(deg) = \frac{\vartheta_F}{\sigma_{max}} = \frac{k\sqrt{\varepsilon_m}Re[\Delta\alpha_B]}{\sigma_{max}}$

where α_B and $\Delta\alpha_B$ are calculated according to Equation S 4 and Equation 4 (main text), for an applied field of 1 Tesla. The Drude dielectric function is used for the NCs:[3]

Equation S 9 $\varepsilon(\omega) = \varepsilon_\infty - i\frac{\omega_p^2}{\omega}\frac{\gamma-i\omega}{\omega^2+\gamma^2} = \varepsilon_\infty - i\frac{e^2N}{m\varepsilon_0\omega}\frac{\gamma-i\omega}{\omega^2+\gamma^2}$

where ε_∞ is the background permittivity of the semiconductor NC, γ is the electron damping parameter, N and $m*$ are the free electron density and effective mass, ε_0 is the vacuum permittivity, e is the electron charge, ħ the barred plank constant, and $\omega_P = \sqrt{\frac{e^2N}{m\varepsilon_0}}$ is the plasma frequency.

For the helicity-dependent polarizability, Equation S 10 is used for the coupling function of the oxide, following the Drude formulation reported by Gu and Kornev,[1,2] while a f_m of $1.06\cdot10^{-6}$ is used for the solvent.[2]

Equation S 10 $f(\omega) = -\frac{e^3N}{m^2\varepsilon_0\omega}\frac{(\gamma-i\omega)^2}{(\gamma^2+\omega^2)^2}$

Equation S 9 and Equation S 10 are inserted into Equation S 4 and then into Equations S5-S8, to obtain the fitting functions. Using these equations, the normalized extinction, ellipticity and rotation spectra were fitted simultaneously, using N, m and γ as common parameters for the three fitting functions. ε_m of the solvent (see section S3) was used in Equation S 4, while values of ε_∞ of 4.0 and 5.6 were used respectively for ITO and

FICO NCs, taken from the literature.[7,8] N, $m*$ and γ were obtained from the fitting. For ITO NCs a frequency dependent damping function ($\gamma(\omega)$, Equation S 11) was used to account for the strong electron scattering caused by ion impurities,[7,9] while for FICO NCs, a constant value of γ was sufficient to fit the spectra.

Equation S 11 $$\gamma(\omega) = \gamma_L - \frac{\gamma_L - \gamma_H}{\pi}\left[\tan^{-1}\left(\frac{\omega - \omega_X}{\omega_W}\right) + \frac{\pi}{2}\right]$$

Indeed, dopant impurities are known to give rise to electron-scattering which is generally rationalized with the presence of two damping regimes: one at high frequency, characterized by a damping constant γ_H, where the electrons can escape the Coulomb interaction with the impurity ions, as they travel faster; and one at low frequency, characterized by γ_L, where the damping is higher.[9,10] The two regimes are separated by a frequency threshold ω_X, while the width of the crossing region between the two regimes is expressed by ω_W. The different behaviour between ITO and FICO can be explained with the fact that Sn^{4+} ions scatter electrons more efficiently than In^{3+},[11] due to the higher charge. In addition, the electron scattering is negligible for the F^- co-dopant in FICO, due to its lower charge with respect to the Sn^{4+} and In^{3+} impurities.[12] Alternative fitting approaches reported in the literature reproduced with good agreement the asymmetric line shape of the LSPR in ITO NCs by considering a distribution of carrier densities.[7] Employing the latter model similar agreement with the experimental extinction and MCD was obtained (not reported here), as well as comparable free carrier parameters.

Parameters obtained from the fitting are reported in Table S1, while the $\gamma(\omega)$, used for ITO is reported in Figure S 2C and Table S 2. The fitting curves obtained with this approach, employed on the three experimental spectra, are the one reported in Figure 3 of the main text.

Alternatively, one can fit simultaneously only extinction and ellipticity spectra (Figure S2 for ITO and Figure S3 for FICO NCs), obtaining similar agreement with the experimental curves, as well as retrieving comparable free electron parameters. In some cases this latter double simultaneous fit (Extinction and Faraday ellipticity) may be preferrable as the measurement of Faraday rotation can be challenging especially for the subtraction of the solvent contribution.

Table S 1: LSPR parameters and Drude parameters obtained from the analysis of the extinction and ellipticity spectra.

	λ_{LSPR} *(nm)* [a]	*FWHM (eV)* [a]	*N* (cm^{-3}) [b]	*m* [b]	ω_c *(meV/Tesla)* [c]	ω_c/γ *x1000* (T^{-1}) [d]
Au	520	0.410	5.57 x 10^{22}	$0.99m_e$	0.114	0.28
ITO	1825	0.190	7.00 x 10^{20}	$0.26m_e$	0.444	2.33
FICO	1883	0.103	8.96 x 10^{20}	$0.30m_e$	0.384	3.84

a) obtained from the analysis of the extinction spectrum; b) obtained from the simultaneous fit of extinction and ellipticity spectra. Data for Au are taken from references;[20,39] *c) cyclotron frequency calculated from electron effective mass using the following relation:* $\omega_c = eB/m$*, calculated for 1 Tesla of applied field. d) magnetoplasmonic quality factor calculated as* ω_c/γ*.*

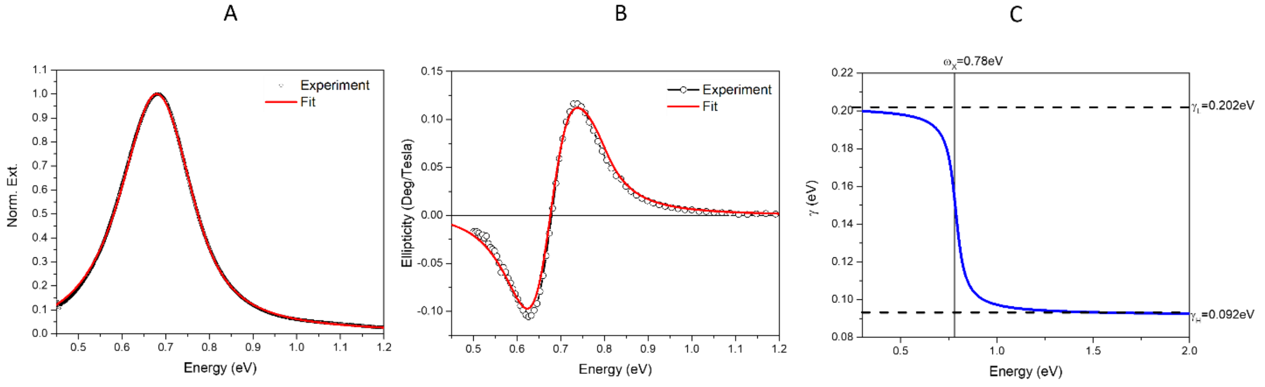

Figure S 2: Fitting of extinction (A) and Faraday ellipticity (B) spectra of ITO NCs. (C) Frequency dependent damping parameter retrieved from the fitting.

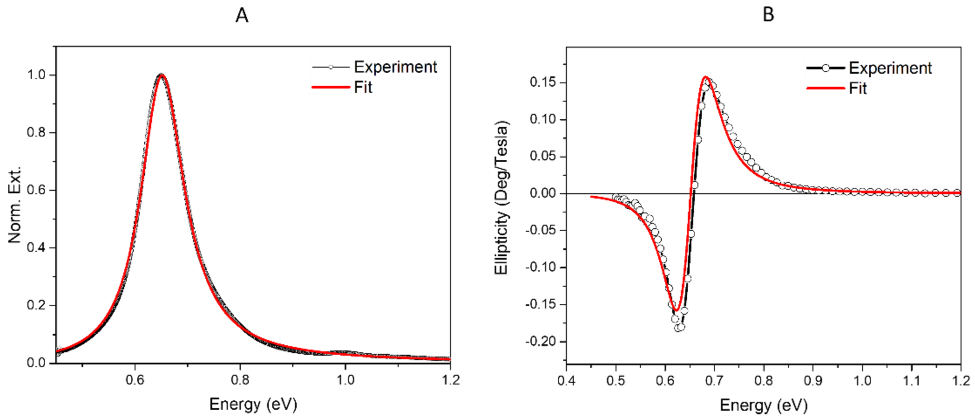

Figure S 3: Fitting of extinction (A) and Faraday ellipticity (B) spectra of FICO NCs.

Table S 2: Damping parameters of obtained from the fit of extinction and MCD spectra for ITO and FICO NCs.

	γ_L **(eV)**	γ_H **(eV)**	ω_x **(eV)**	ω_w **(eV)**
**ITO**	0.202	0.092	0.78	0.035
**FICO**	0.102	-	-	-

S4. Sensing experiments

The refractive index of the solvents used in the main text were taken from the literature.[13,14] As can be seen in Figure S5, the dispersion curve is quite flat in the region of interest (1200-2400 nm) for all the three solvents, which makes reasonable for us to take a constant value for the refractive index of the solvent. Values of 1.4477, 1.4895 and 1.5866 are used for CCl_4, C_2Cl_4 and CS_2, respectively.

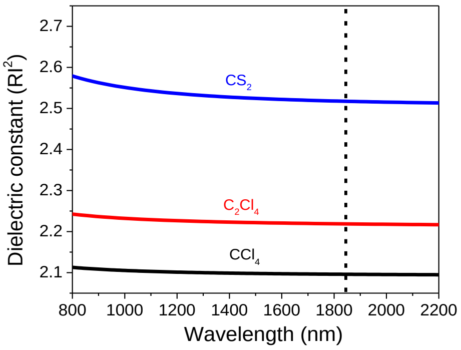

Figure S 4: Dielectric constant vs wavelength curve for the solvent used in this work. Data from Kedenburg *et al.*[13] are used for CCl_4 and CS_2, while data from Chemnitz *et al.*[14] are used for C_2Cl_4. The dashed vertical line indicates the region of the LSPR peak for our samples.

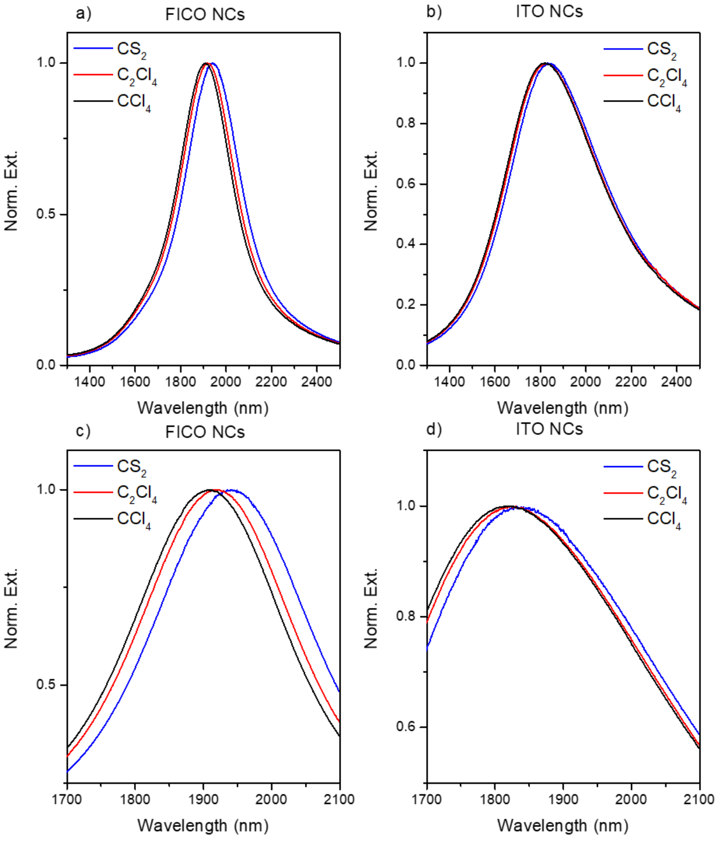

Figure S 5: Normalized extinction spectra of FICO (a) and ITO (b) NCs dispersed in three different solvents with different refractive index and magnification in the region of the LSPR maximum c, d).

S5. Comparison of magnetoplasmonic performances

Table S 3 Comparison of magnetoplasmonic performances of ITO and FICO NCs with the most promising systems reported in the literature.

	**Au NPs (i)**	**Ni (ii)**	**Ni array (iii)**	**Au/SiO2/Ni array (iii)**	**ITO NCs (iv)**	**FICO NCs (iv)**
**a) λ_{LSPR} (nm)**	520	650	800	870	1825	1880
**b) $\Delta\lambda/\Delta RI$ (nm/RIU)**	80	250	214	211	184	223
**c) $\Delta\varepsilon/\Delta RI$ (deg/RIU)**	0.029	0.50	0.44	0.63	0.29	1.24
**d) $\Delta\theta/\Delta RI$ (deg/RIU)**	N.A.	N.A.	0.64	0.89	0.22	1.12
**e) B (Tesla)**	1.3	0.4	0.4	0.4	1.4	1.4

a) position of the LSPR peak in extinction; b) refractive index sensitivity of the LSPR extinction peak; c,d) refractive index sensitivity of the magneto-optical signal (ellipticity and rotation); e)Applied field employed in the experiments.(i) Data from Reference 2. (ii) Data from reference 15. (iii) Data from reference 16. (iv) Data from the present work.

References

(1) Gu, Y.; Kornev, K. G. Plasmon Enhanced Direct and Inverse Faraday Effects in Non-Magnetic Nanocomposites. *J. Opt. Soc. Am. B, JOSAB* **2010**, *27* (11), 2165–2173. https://doi.org/10.1364/JOSAB.27.002165.

(2) Pineider, F.; Campo, G.; Bonanni, V.; de Julián Fernández, C.; Mattei, G.; Caneschi, A.; Gatteschi, D.; Sangregorio, C. Circular Magnetoplasmonic Modes in Gold Nanoparticles. *Nano Letters* **2013**, *13* (10), 4785–4789. https://doi.org/10.1021/nl402394p.

(3) Kreibig, U.; Vollmer, M. *Optical Properties of Metal Clusters*; Toennies, J. P., Gonser, U., Osgood, R. M., Panish, M. B., Sakaki, H., Lotsch, H. K. V., Series Eds.; Springer Series in Materials Science; Springer Berlin Heidelberg: Berlin, Heidelberg, 1995; Vol. 25.

(4) Sepúlveda, B.; González-Díaz, J. B.; García-Martín, A.; Lechuga, L. M.; Armelles, G. Plasmon-Induced Magneto-Optical Activity in Nanosized Gold Disks. *Physical Review Letters* **2010**, *104* (14). https://doi.org/10.1103/PhysRevLett.104.147401.

(5) Floess, D.; Weiss, T.; Tikhodeev, S. G.; Giessen, H. Lorentz Nonreciprocal Model for Hybrid Magnetoplasmonics. *Physical review letters* **2016**, *117* (6), 063901. https://doi.org/10.1103/PhysRevLett.117.063901.

(6) Zvezdin, A. K.; Kotov, V. A. *Modern Magnetooptics and Magnetooptical Materials*; Studies in condensed matter physics; Institute of Physics Pub: Bristol : Philadelphia, Pa, 1997.

(7) Mendelsberg, R. J.; Garcia, G.; Li, H.; Manna, L.; Milliron, D. J. Understanding the Plasmon Resonance in Ensembles of Degenerately Doped Semiconductor Nanocrystals. *J. Phys. Chem. C* **2012**, *116* (22), 12226–12231. https://doi.org/10.1021/jp302732s.

(8) Kriegel, I.; Urso, C.; Viola, D.; De Trizio, L.; Scotognella, F.; Cerullo, G.; Manna, L. Ultrafast Photodoping and Plasmon Dynamics in Fluorine–Indium Codoped Cadmium Oxide Nanocrystals for All-Optical Signal Manipulation at Optical Communication Wavelengths. *J. Phys. Chem. Lett.* **2016**, *7* (19), 3873–3881. https://doi.org/10.1021/acs.jpclett.6b01904.

(9) Hamberg, I.; Granqvist, C. G. Evaporated Sn-doped In2O3 Films: Basic Optical Properties and Applications to Energy-efficient Windows. *Journal of Applied Physics* **1986**, *60* (11), R123–R160. https://doi.org/10.1063/1.337534.

(10) Lounis, S. D.; Runnerstrom, E. L.; Llordés, A.; Milliron, D. J. Defect Chemistry and Plasmon Physics of Colloidal Metal Oxide Nanocrystals. *J. Phys. Chem. Lett.* **2014**, *5* (9), 1564–1574. https://doi.org/10.1021/jz500440e.

(11) Mendelsberg, R. J.; Zhu, Y.; Anders, A. Determining the Nonparabolicity Factor of the CdO Conduction Band Using Indium Doping and the Drude Theory. *J. Phys. D: Appl. Phys.* **2012**, *45* (42), 425302. https://doi.org/10.1088/0022-3727/45/42/425302.

(12) Ye, X.; Fei, J.; Diroll, B. T.; Paik, T.; Murray, C. B. Expanding the Spectral Tunability of Plasmonic Resonances in Doped Metal-Oxide Nanocrystals through Cooperative Cation–Anion Codoping. *J. Am. Chem. Soc.* **2014**, *136* (33), 11680–11686. https://doi.org/10.1021/ja5039903.

(13) Kedenburg, S.; Vieweg, M.; Gissibl, T.; Giessen, H. Linear Refractive Index and Absorption Measurements of Nonlinear Optical Liquids in the Visible and Near-Infrared Spectral Region. *Opt. Mater. Express, OME* **2012**, *2* (11), 1588–1611. https://doi.org/10.1364/OME.2.001588.

(14) Chemnitz, M.; Gaida, C.; Gebhardt, M.; Stutzki, F.; Kobelke, J.; Tünnermann, A.; Limpert, J.; Schmidt, M. A. Carbon Chloride-Core Fibers for Soliton Mediated Supercontinuum Generation. *Opt. Express* **2018**, *26* (3), 3221. https://doi.org/10.1364/OE.26.003221.

(15) Maccaferri, N.; E. Gregorczyk, K.; de Oliveira, T. V. A. G.; Kataja, M.; van Dijken, S.; Pirzadeh, Z.; Dmitriev, A.; Åkerman, J.; Knez, M.; Vavassori, P. Ultrasensitive and Label-Free Molecular-Level Detection Enabled by Light Phase Control in Magnetoplasmonic Nanoantennas. *Nature Communications* **2015**, *6*, 6150. https://doi.org/10.1038/ncomms7150.

(16) Pourjamal, S.; Kataja, M.; Maccaferri, N.; Vavassori, P.; Van Dijken, S. Hybrid Ni/SiO2/Au Dimer Arrays for High-Resolution Refractive Index Sensing. *Nanophotonics* **2018**, *7* (5), 905–912.
